# Development of Laser Drilling Strategy for Thick Carbon Fibre Reinforced Polymer Composites (CFRP)

**DOI:** 10.3390/polym12112674

**Published:** 2020-11-12

**Authors:** Sharizal Ahmad Sobri, Robert Heinemann, David Whitehead

**Affiliations:** 1 Advanced Material Research Cluster, Faculty of Bioengineering and Technology, Universiti Malaysia Kelantan, Jeli Campus, Jeli 17600, Kelantan, Malaysia; 2 Department of Mechanical, Aerospace and Civil Engineering, The University of Manchester, Sackville Street Building, Sackville Street, Manchester M13 9PL, UK; robert.heinemann@manchester.ac.uk (R.H.); david.whitehead@manchester.ac.uk (D.W.); 3 Geopolymer and Green Technology, Center of Excellence (CEGeoGTech), Universiti Malaysia Perlis, Kangar 01000, Perlis, Malaysia; 4 Global Entrepreneurship Research and Innovation Centre (GERIC), Universiti Malaysia Kelantan, City Campus, Kota Bharu 16100, Kelantan, Malaysia

**Keywords:** drilling method, machining parameters, laser drilling, carbon fibre reinforced polymer composite (CFRP), hole depth, heat-affected zone (HAZ)

## Abstract

Composites from carbon fibre reinforced polymers (CFRPs) play a significant role in modern manufacturing. They are typically used in aerospace and other industries that require high strength-to-weight ratios. Composite machining, however, remains a challenging job and sometimes is hampered by poor efficiency. Despite considerable research being conducted over the past few years on the machining of composite materials, the material nevertheless suffers from delamination, fibre loss, and imperfect finishing of the fuselage. Laser technology is becoming increasingly popular as an alternative approach to cutting and drilling composites. Experiments have been conducted with a CFRP thickness of 25.4 mm using fibre laser to test the effect of the machining parameters on the primary performance measurements. In this study, different machining criteria are used to assess the fibre laser ability of thick CFRP composites for drilling operation. The experimental findings revealed that a fibre laser is capable of penetrating a thick CFRP to a depth of 22 mm by using a novel drilling procedure.

## 1. Introduction

The use of advanced composite materials influences aircraft design and construction, and about 50% of overall aircraft production costs are incurred [1,2]. The manufacturability of such composite products depends, among other things, on the ease with which the materials can be produced in the required shape [3]. However, regardless of the forming process, according to Hintze et al. [4] a post-machine step, such as milling and drilling, is necessary after curing to create the shape with the required tolerances. For components made of carbon fibre reinforced polymers (CFRP), common problems that occur during manufacturing like fibre pull-outs, delamination, and putrefaction of the material matrix can affect the consistency and properties of machined surfaces [1,4,5,6,7]. Therefore, there is an increasing interest in composite machining, particularly to reduce the damage caused by different machining processes.

CFRP machining work has concentrated almost exclusively on CFRP sections of below 25 mm in thickness, and there are few references to the drilling of thick CFRP composites of about one inch or 25 mm. Nevertheless, aircraft companies are now more involved in the machining of thick CFRP, as CFRP has begun to move to an aircraft’s high-load areas where thick wall components are needed. CFRP is a hard-to-machine material for discontinuity, inhomogeneity and anisotropic design. It is safe to expect that the machining of increasingly thick CFRPs would be more challenging in terms of machining operations (e.g., mechanical drilling, for instance) due to the prolonged tool engagement time and the increasingly difficult swarf/chip evacuation. Additionally, an increased thickness in the case of laser drilling leads to a more extended period of contact between the laser beam and material, which results in different vaporising effects. In addition to research on conventional machining, there has also been a steady rise in research on non-conventional composite machining. For example, laser and water jet cutting, electro discharge, electrochemical and ultrasonic machining are examples of unconventional technology. Laser machining tends to be a feasible solution since one of the composites is always a polymer form, and polymers usually have a high infra-red coefficient of radiation and low thermal conductivity, resulting in a high level of thermal energy [8,9].

This article examines the laser machining of CFRP composites by drilling small diameter holes (i.e., 8 mm) into 25.4 mm-thick CFRP composites. The main objective was to investigate the relationships between process parameters (laser power, scanning speed, focal plane position (FPP), assist gas pressure, modulated laser beam for laser machining) and hole quality (delamination, fibre pull out, heat-affected zone (HAZ), hole dimensional accuracy and other damages) to identify the process parameters that affect cut quality in laser drilling operations. The second objective was to optimise the laser drilling process to reduce the defects during the fabrication of boreholes.

## 2. An Overview of the Effect of Laser Machining Parameters

Abrate and Walton [9] emphasise that laser cutting is challenging as component thickness increases, because the layer being extracted prevents the laser beam from cutting. The laser cutting of plastics is very effective because of the high absorption rate and low heat conductivity of infrared radiation, which keep the thermal energy highly localised [10]. The following explanations are from pioneering studies on carbon fibre reinforced composites, and most focus on CO_2_ (carbon dioxide) and Nd: YAG (neodymium-doped yttrium aluminum garnet) lasers, which are widely used in composite machining. In contrast to these two lasers, there were fewer references relating to fibre lasers. Additionally, the main issues about the application of process parameters (i.e., laser power, cutting speed, type of gas and its pressure and material thickness on the quality of the workpiece) have been considered by other researchers who used CO_2_, Nd: YAG, and other types of lasers.

Reza and Li [11] examined the use of 1 kW fibre laser IPG YLR-1000-SM for laser cutting in carbon fibre reinforced polymer composites at the University of Manchester’s Laser Processing Research Centre (LPRC). The thermal damage caused by various gases and pressure assisting forms, as well as focal plane positions (FPP), were investigated through experimental studies. They concluded that the essential factors in single-pass mode are beam power and scanning speed. Based upon the analysis, the results of the FPP scan showed that these optimal conditions successfully reduced CFRP in a one-pass procedure under the surface of the workpiece with a laser power of 340 W and a scan speed of 20 mm/s. The fibre laser system used a beam focused below the material for the cutting experiments, and which, according to the authors, was used for thermal damage reduction. They noted that even at low scanning rates of 1 mm/s, the material in the piece could not be cut at power levels below 230 W. Furthermore, Lau et al. [3] reached the same verdict as Reza and Li [11]. They stated that it is essential to understand the interaction between a laser beam and workpiece because laser cutting quality is strongly influenced by laser power and cutting speed/feed rate, as highlighted by Klotzbach et al. [12].

Lau et al. [3] conducted the experiments on the performance of Nd: YAG laser and Excimer laser on the machining of CFRP. Their results indicate that intermittent cutting produces an inferior surface finish, and that scanning speed is vital. Klotzbach et al. [12] conducted laser machining of CFRP experiments by using three high brilliant laser beam sources—a single-mode fibre laser, a multi-mode fibre laser and CO_2_ slab laser. They identified that the number of cycles plays a significant role in terms of HAZ reduction when increasing the laser spot velocity during the remote processing. The higher the velocity applied, the higher the required cyclic rate to reach the desirable kerf width. Based on these attempts [3,11,12], each combination of laser power and speed produces significantly different cutting quality. For instance, as high power and low speed is applied, it may effectively remove the material in large amounts, but at the same time, it creates significant HAZ due to high energy of the laser and the interaction time between the laser beam and workpiece. Based on previous research, the typical range for laser power is between 10 W and 3 kW, and the range for scanning speed is between 1 and 1000 mm/s.

The effect observed by Reza and Li [11] of the assist gas shows a decrease in the recession in the matrix and the kerf width, i.e., at the entry and exit sides. This result occurred when inert gasses such as argon and nitrogen were used. However, in the case of oxygen, by increasing the gas pressure, the matrix recession can be reduced while the kerf width increases on both sides as the gas pressure increases. They argued that this was due to an oxidation process that caused accelerated decomposition or vaporisation of the material. Mathew et al. [13] conducted parametric studies on pulsed Nd: YAG lasers with a 300 W capacity on machining 2 mm-thick CFRP, by studying the HAZ and the kerf taper as their outcomes. They used argon and found that it provided a positive laser machining performance, similar to the results of Reza and Li [11].

The next observation made by Reza and Li [11] is the effect of FPP. The range was set at −2.38 mm to +2.38 mm with a constant 340 W laser power, 20 mm/s scanning speed and 8 bar gas pressure. As the FPP slowly increased from −2.38 mm, the results show that heat damage occurred at the entrance to the beam, while the thermal damage decreased at the end of the cutting depth. Furthermore, as it reached 2.38 mm, this upward push triggered incomplete cuts. Based on the above results, increasing the FPP can affect the surface power density, as well as the change in the beam size. The effect of FPP influences the material’s thermal impact. However, this test was performed on 2 mm CFRP, and the adjustment of FPP is assumed to vary because of thickness problems related to changes in power densities during FPP transition. A smaller beam spot creates an energy density against the material that is efficient and highly oriented. Focus consistency and energy distribution stability allow the laser beam spot to expand the surface quality in the machining. This experiment also indicated the superiority of a fibre laser’s beam focusing position compared to the experiments of CFRP composites machining by using CO_2_ and Nd: YAG lasers [14].

The laser cutting of CFRP by Nd: the YAG laser was examined by Wahab et al. [14]. They concluded that the pulse Nd: YAG laser can be effectively reduced to 30 mm/m with a pulse duration of 0.5 ms. Furthermore, the significant effects on kerf width, HAZ and taper angle are affected by the pulse energy and the pulse repetition rate, where a high pulse energy leads to a high HAZ, and a high pulse repetition rate produces a narrow kerf width. As the authors suggested, laser cutting plays a significant role in pulse energy and repetition rate. However, given the fact that the authors were able to attain the optimal parameters, they succeeded in reducing a 1.5 mm thickness of CFRP, which is challenging. This result shows that the operation of pulse mode needs more parameters, including pulse duration, pulse energy and pulse repetition rate to optimise the laser beam efficiency and power density. Additionally, full penetration cuts are almost impossible due to the available energy supplies during the laser machining process. The optimum parameters, such as short contact time and more effective laser power, must be sought for future study. Those are the key criterion for achieving the best level of reductions. Additional parameters not included in pulse manipulation can also increase the surface quality and save time when penetrating the hole area. The difficulty of using laser pulse-mode reinforces the superiority of fibre lasers in CFRP machining and is evident in these studies [3,13]. The usual range of pulse off-time is between 1 and 90 ms, while the pulse repetition rate is between 0.5 and 20 kHz. Thus, Table 1 lists the most critical parameters for laser machining.

## 3. Materials and Methods

### 3.1. Materials

The material samples with the size of 50 (length) × 20 (width) mm used for the experiments is a multi-directional multi-layer carbon fibre reinforced composite, with an overall thickness of 25.4 mm (1 inch), supplied by Airbus UK, Broughton. A visual inspection showed that the lamina’s stacking series was arranged to a thickness of up to 25.4 mm [0°/90°/−45°/90°/45°/90°/−45°/90°/45°/90°/−45°/90°/0°]. Each ply has a thickness of 0.22 mm. A Keyence Digital VHF-500X digital optical microscope was used to capture the damage occurrence at entry and exit holes, including inside the hole. The matrix or bonding agent is thought to be epoxy. The properties of CFRP material are shown in Table 2.

### 3.2. IPG 1 kW Fibre Laser and Clamping Device for Laser Experiments

**Figure 1:**
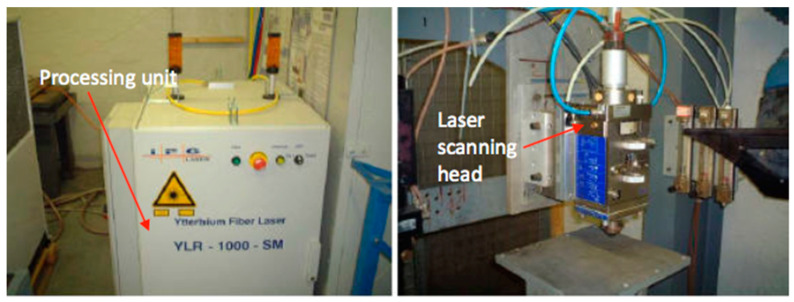
IPG single-mode YLR-1000-SM 1 kW fiber laser.

**Figure 2:**
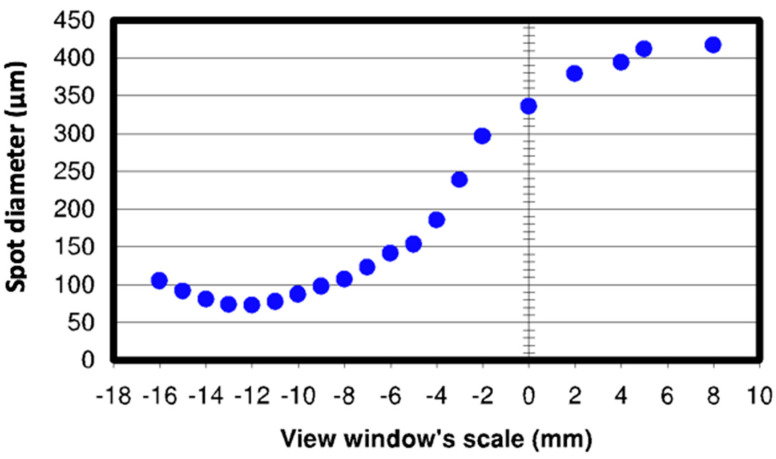
Variation of Ø spot sizes overview window’s scale for Precitec HP1.5” (adapted from Reza [15]).

**Figure 3:**
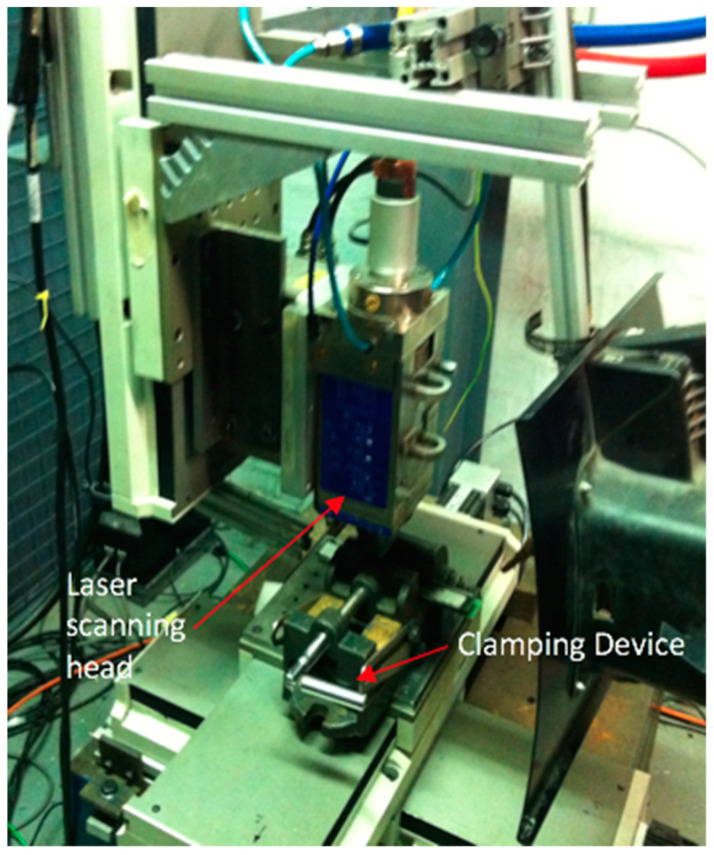
Clamping device was set on the laser machining table.

The IPG single-mode YLR-1000-SM (IPG Photonics (UK) Ltd., Bristol, UK) (see Figure 1) laser machining system used for the machining tests. It is a single-mode configuration of 1 kW Ytterbium-doped with a wavelength of nearly infrared 1070 nm. It has a beam quality of M2 = 1.1 and functions with frequencies of up to 1 kHz in either the continuous-wave (CW) or the modulated pulsed mode. The focal lens is 38 mm in diameter, 190 mm long, and has a minimum focused spot size of 70 µm. The target position can be changed manually (i.e., view window range −20 to +10 mm). The range of focused spot sizes on the viewing window is shown in Figure 2. A linear drive operated with the Precitec MC870 motor control, for vertical positioning, is built into this laser motor. The laser device is built into a linear PC controlled motor stage, which allows for shifting the workpiece underneath the fixed cutting head in the *x*-*y* direction. The clamping device, as it was inside the operating room of the laser, is shown in Figure 3.

### 3.3. Experimental Setup

**Figure 4:**
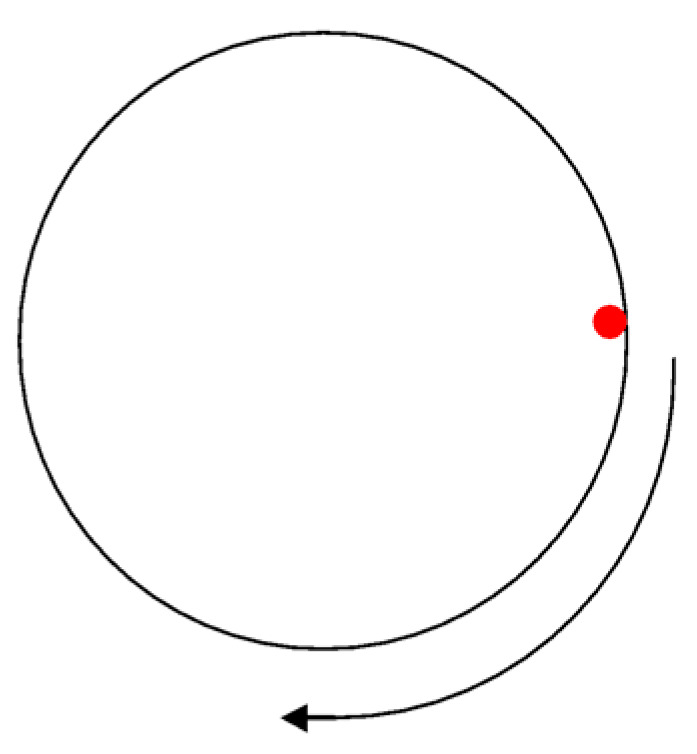
Laser drilling movement.

Laser drilling of 25.4 mm-thick CFRP was carried out on the periphery of each hole using laser beam scanning. Two holes were drilled with a diameter of 8 mm on each sample block, which had a similar arrangement to that of mechanical drilling. The first process window for drilling thick CFRP focused on the laser power, varying between 400 and 900 W in continuous mode (CW) operation. The other parameters were constant and as follows: scanning speed of 10 mm/s; argon assist gas; gas pressure at 8 bar; nozzle Ø 1 mm; 1 mm stand-off distance; focal plane position (FPP) at −12 (equivalent to a beam spot diameter, 70 µm). The experiments were replicated twice to ensure the reliability of the machining centre, and the number of samples was limited to avoid wastage and to prepare for other experiments. Each hole was drilled at the circumferential outline, and 10 passes were used, since the single-pass was unable to drill 25.4 mm thick CFRP. Figure 4 shows the drilling movement, where the laser beam starts and ends at the same red dot point and moves in a clockwise pattern. The purpose of this experiment was to identify a potential laser power to meet the challenge of drilling the unusually thick material.

**Figure 5:**
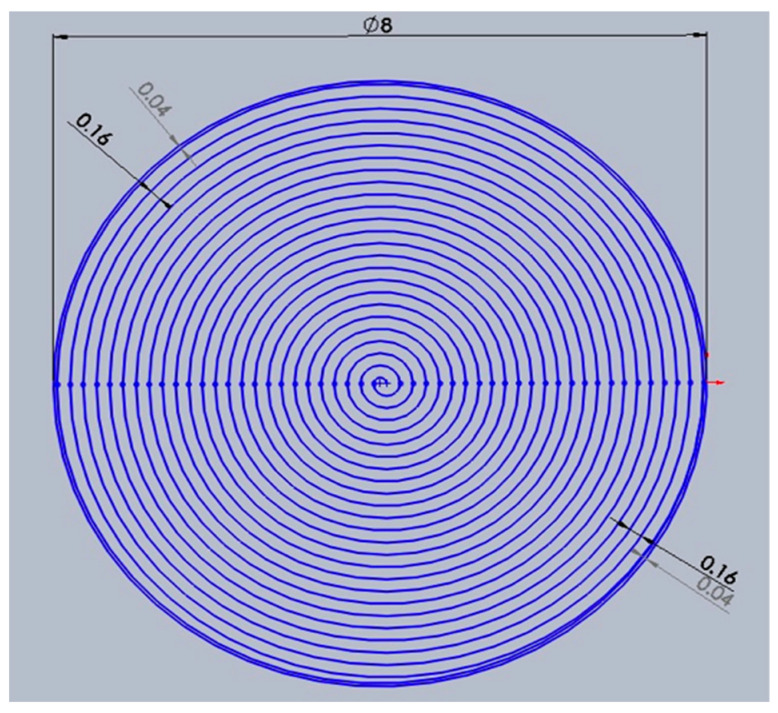
Spiral trepanning.

The next experimental study was to improvise the drilling movement from a ring shape to a spiral hole cutting shape. Additionally, the spiral trepanning has not been attempted yet by any researchers for drilling thick CFRP (i.e., more than 9 mm). Figure 5 shows the design of the laser drilling movement in a spiral pattern (all units are in mm), in which the laser beam starts at the centre point of a hole, then moves until it ends at the edge of a hole as indicated by the red arrow. The gap between each spiral path is 0.16 mm. In contrast, the gap adjacent to the surface edge is 0.04 mm to reduce the amount of laser energy being bombarded directly on the surface, which may reduce the HAZ or other potential damage. The process parameters for the next observations are shown in Table 3. All parameters are constant except for the laser power and scanning speed, where one variable change but all other variables in the experiment are fixed.

A third experiment studied the effect of FPP on the laser machining of CFRP. Research on the effect of focal plane position or FPP is still lacking. However, it can be considered as an additional parameter in finding a new path for drilling thick material; investigation of FPP is necessary to identify the optimal distance between the laser beam and workpiece material for complete penetration. Additionally, this research reports for the first time the influence of FPP on drilling of 25.4 mm-thick CFRP. Because of the discovery of the significant influence of drilling strategy, this experiment investigates the optics system of the IPG single-mode 1 kW fibre laser to find the optimum solution to utilise laser energy efficiently during the removal process.

This investigation was applied to the spiral trepanning strategy based on the preliminary results by Sobri et al. [16] in single pass cutting, due to the positive results achieved in terms of significant material removal rate, and the significant reduction of HAZ and other defects. It is assumed that changing the FPP varies the laser energy level in the machining of CFRP and that this alteration determines the laser beam’s spot size on the surface. Due to the influence of beam spot size, another assumption is that FPP is a possible factor to affect the taper or inclination of cylinder-shaped holes. All process parameters were set at constant values except for the variation of FPP, where four views of the window’s scale were selected at −16, −14, −10 and −8, excluding the reference point at −12 because the spiral trepanning strategy made it feasible to drill more than 5 mm depth of cut at this point. Other process parameters were as follows: P = 900 W; scanning speed = 10 mm/s; the assist gas type was argon with pressure set at 8 bar; a 1 mm Ø nozzle size; with a 1 mm stand-off distance.

A fourth experiment studied the effect of assist gas pressure on the hole depth, HAZ and hole diameter. Moreover, as highlighted in the literature review, increasing the assist gas pressure can reduce thermal damage to the material, and it is considered to be an influential factor for the depth of cut. However, most previous attempts were only applied to materials with a thickness of up to 7 mm. Constant parameters were 900 W laser beam power, 10 mm/s scanning speed, −12 FPP, argon gas type, Ø 1 mm for the nozzle and 1 mm stand-off distance. The variable parameter was assisted gas pressure which varied from 8, 7, 6 and 5 bar. The effect of assist gas pressure is studied in this section without considering variations of gas type because argon gives the best quality appearance, and acts mechanically to remove the vaporised material.

**Figure 6:**
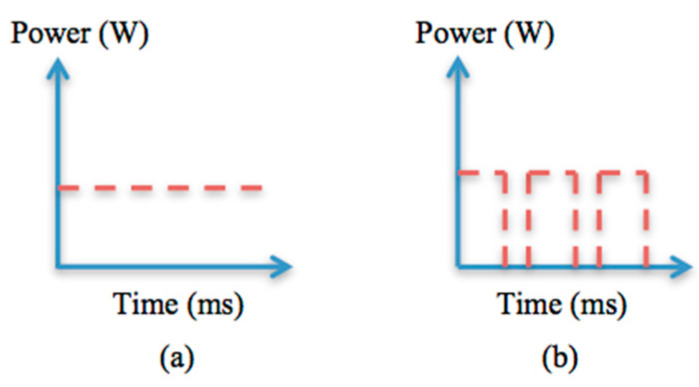
Energy delivery pattern: (**a**) continuous wave; (**b**) modulated pulse wave.

Previous research agreed that laser machining in pulse mode is an alternative way of supplying laser energy efficiently into the workpiece material, and it is a well-recognised approach for heat-sensitive material like CFRP. Even though there is a limitation of cutting depth for a complete hole, it is worthwhile attempting this approach to drill 25.4 mm-thick CFRP. The IPG 1 kW single-mode fibre laser system delivers a CW beam or a modulated pulsed beam at frequencies of up to 1 kHz. Figure 6 shows different patterns of energy delivery, and the modulated pulsed beam can be programmed for such delivery patterns. The experimental study on modulated pulse mode is discussed in this sub-section. The same parameters as in Table 3 were used, but with some changes, as shown in Table 4. Variable parameters (i.e., pulse-off time and pulse repetition rate) were selected based on a range of characteristic parameters applied in previous research. A spiral trepanning drilling strategy with single-pass cutting was applied in this study to identify whether it is possible to achieve full penetration. 

## 4. Results and Discussion

**Figure 7:**
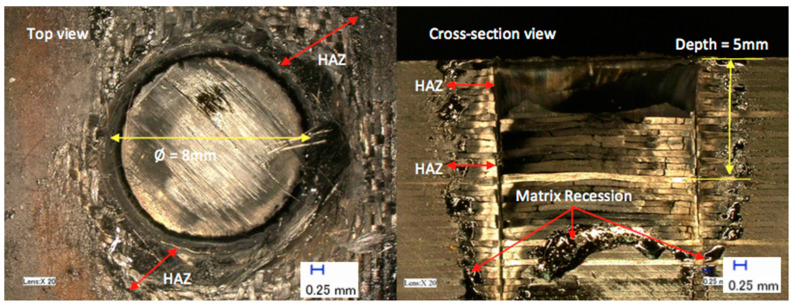
Typical example of a hole at a laser power of 600 W.

**Figure 8:**
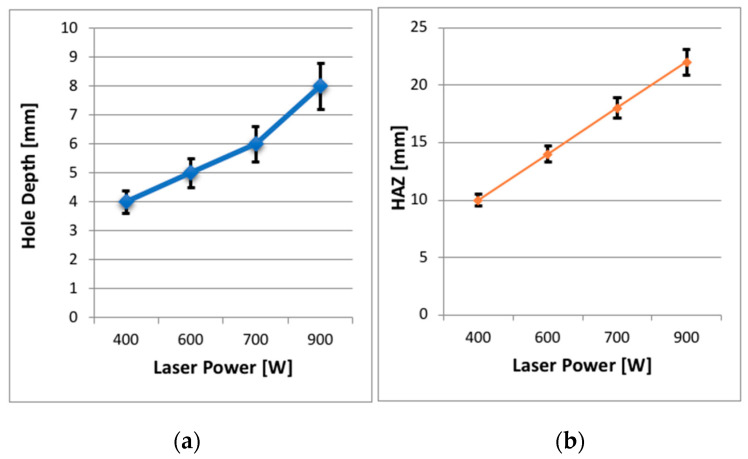
Effect of laser power at a constant scanning speed of 10 mm/s on: (**a**) hole depth; (**b**) heat-affected zone (HAZ).

Figure 7 shows the effect of laser power on a hole depth and HAZ, while Figure 8 displays the laser power results. Figure 7 shows that the HAZ and matrix recession also occurred on the workpiece in this study. The interaction between laser and workpiece was the same as before (i.e., energy pumped into the material) with the only difference being that the laser passed over the material multiple times (i.e., 10 passes), which meant the material was exposed to the laser for longer. The measurement of each hole depth was repeated three times for all experiments, and the average depth was calculated. Figure 8a shows a linear relationship between laser power and the hole depth in that an increased laser power brought about an increase in the depth of the hole. However, the laser beam was not able to produce a complete hole through the entire thickness of the material regardless of the applied laser power, i.e., it could not penetrate the workpiece. A multiple-pass cutting approach was applied, based on suggestions from past researchers for an alternative cutting strategy mechanism to achieve the highest depth and lowest amount of thermal damage. The most significant hole depth was only 8 mm at laser power of 900 W. Even though 10 passes were used, the laser beam did not penetrate through the entire CFRP material. This result indicates that the laser beam is unable to cut because of insufficient cleaning by the assisting gas. Plumes are sometimes left behind, which can prevent the laser beam from reaching the material further down.

It was found that HAZ appeared on the surface adjacent to the hole’s periphery when the power level changed from 400 to 900 W, as shown in Figure 8b. Increasing the laser power is recommended in laser drilling of CFRP in previous research because more energy can be utilised to cut the workpiece. As expected, with the increasing power, the material inside the hole was able to cut deeper. Additionally, the vaporisation temperature of CFRP is much higher than that of aramid, glass or graphite, so a higher temperature was needed to cut the thick CFRP composites [9,17]. Furthermore, since the thermal conductivity of carbon fibre is much higher than the composite materials, much of the heat is conducted away, and the high temperatures remain localised in the cutting zone. This factor may be the reason for the high dissipation of heat into the workpiece, which created a HAZ.

According to the results, the multi-pass approach can be applied for drilling thick CFRP, since the fibre laser can penetrate depths of more than 5 mm. However, the drilling operation revealed that the window where the laser power and scanning speed vary, it cannot penetrate deeper into the material. This could be explained by the limitation of the stand-off distance of the nozzle, where only a 1 mm is possible with the IPG 1 kW fibre laser at present. However, neither the laser power nor scanning speed depends on the stand-off distance. In this case, the stand-off distance that could be achieved was problematic in connection with the focal point plane position and the gas pressure. Concurrently, this limitation ensured that the laser beam retains consistent energy density so that it can reach the material, and retain the efficiency of the gas removal mechanism to reduce the spreading of the gas jet during laser drilling. This is supported by the work of Hernandez et al. [18], who recommend a stand-off distance of 1.5 and double the nozzle exit diameter. On the other hand, for the case of drilling thick CFRP, it is crucial to eliminate evaporated materials effectively.

**Figure 9:**
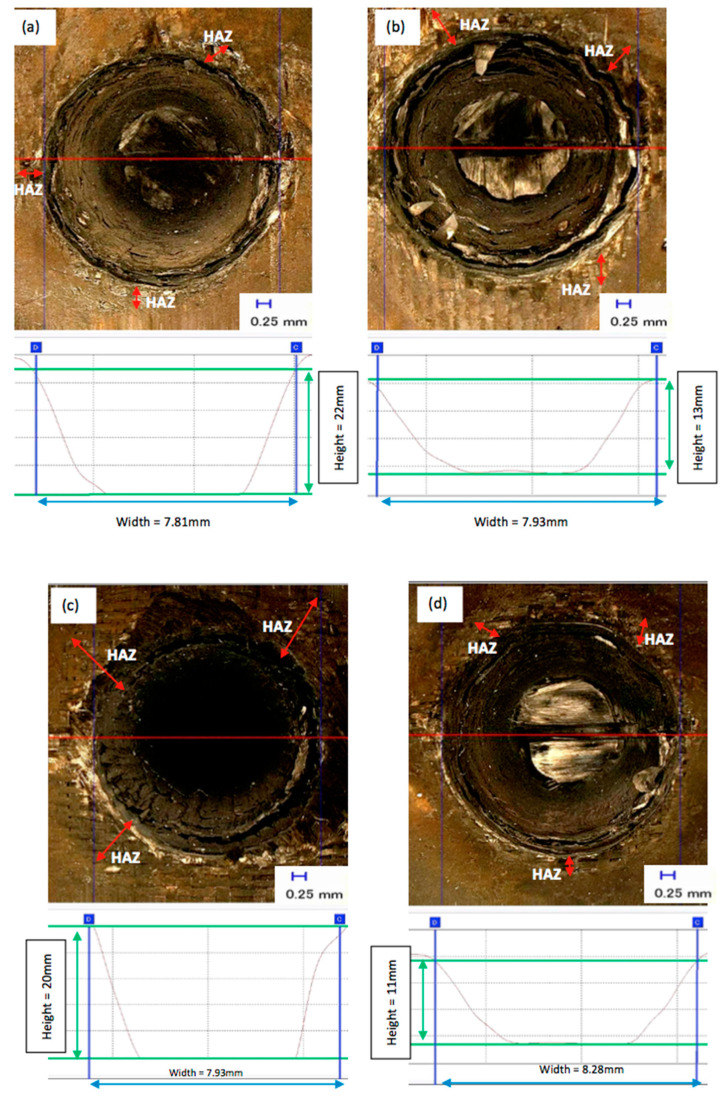
Top view and profile of hole cut at different laser powers/scanning speeds: (**a**) 900 W and (**b**) 400 W at a constant speed of 10 mm/s, and (**c**) with constant laser power of 900 W at 20 mm/s and (**d**) 50 mm/s.

**Figure 10:**
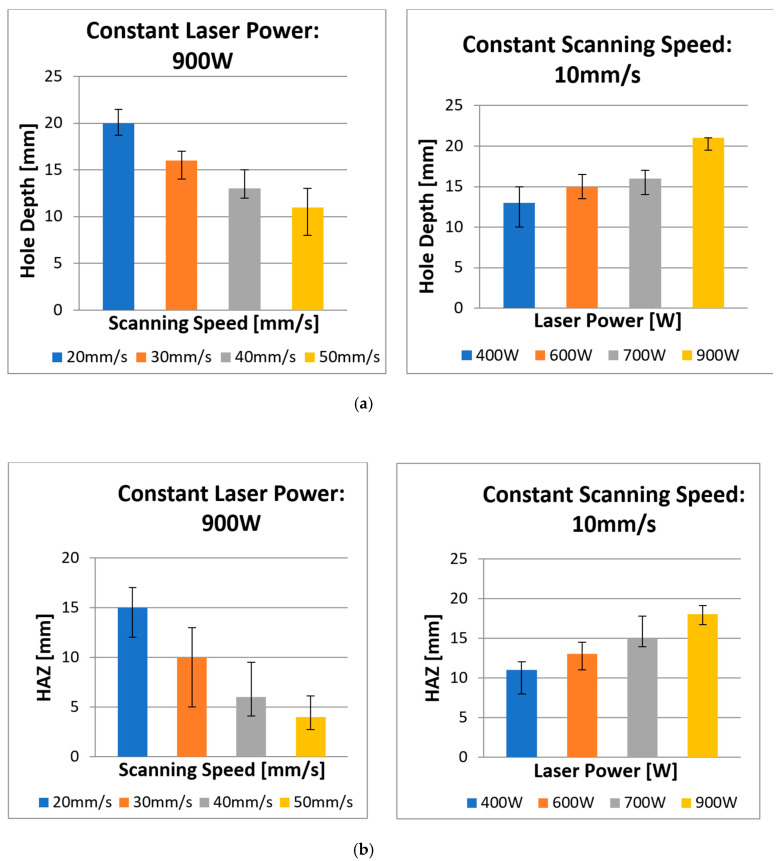
Effect of laser power/scanning speed at a constant speed/power on (**a**) hole depth and (**b**) HAZ.

The present study determines the effects of spiral trepanning strategy with varying laser power and scanning speed to produce a complete hole. In this regard, the process parameters and a graphical illustration of spiral trepanning are contained in Section 3. Figure 9 shows the typical measurement of profile results of laser drilling with the spiral trepanning strategy, and Figure 10 shows the effects of laser power/scanning speed on hole depth and HAZ. In Figure 9, the upper diagrams show the effects of the laser power at the highest (Figure 9a) and lowest (Figure 9b) settings, whereas, the bottom diagrams show the effect of the scanning speed at 20 mm/s (Figure 9c) and at the highest speed of 50 mm/s (Figure 9d). Meanwhile, in both diagrams in Figure 10, the upper diagrams show the relationship between various laser powers/scanning speeds and the hole depth. The bottom diagram indicates the relationship between various laser powers/scanning speeds and HAZ.

Figure 9 and Figure 10 show that there is a linear relationship between the laser power and hole depth/HAZ. When the laser power increased, the hole depth and HAZ also significantly increased. However, there is also an inverse relationship between the scanning speed and hole depth/HAZ, where an increase in scanning speed leads to a reduction in the hole depth/HAZ. Further explanation about the interaction between the laser power and workpiece is similar to the single ring cutting shape in multi-pass results. The positive side of these results is the ability of the fibre laser to penetrate the material in a single-pass with no inland material leftovers, i.e., by-products, inside the hole, which means the material removal is higher than the single ring shape in a multi-passes strategy. Moreover, most hole depths exceeded 7 mm, whereas, with previous strategies, the maximum hole depth was 8 mm. The highest depth of cut was 22 mm and was achieved with the spiral trepanning strategy. Figure 9 shows the majority of the profile shapes showed flat surfaces except for a scanning speed of 50 mm/s, which produced a slightly uneven surface. This result indicates that the spiral movement was able to cut the entire surface more effectively than the single ring cutting shape whereby the laser beam moves along the same path.

Furthermore, the results show that HAZ slightly improved, especially for the variation in cutting speeds, which were significantly reduced by 17%, 44%, 57% and 60% for 20, 30, 40 and 50 mm/s respectively compared to the previous drilling strategy. At a cutting speed of 50 mm/s, there was a significant decrease in thermal damage on the surface adjacent to the circumferential area, including the elimination of matrix recession. The machining time for each scanning speed increased when the spiral trepanning strategy was applied. The machining times for various scanning speeds in a single ring shape with multi-pass strategy (i.e., 10 passes) were 27.22, 16.36, 11.03, 8.43 and 7.23 s for 10, 20, 30, 40, and 50 mm/s respectively. The machining times occurred in spiral trepanning strategy with single-pass were 36.13, 18.79, 12.91, 10.10, and 8.43 s for 10, 20, 30, 40, and 50 mm/s respectively. Each machining time in spiral trepanning strategy was increased by 33%, 15%, 17%, 20%, and 17% for each scanning speed of 10, 20, 30, 40, and 50 mm/s respectively compared to previous drilling strategy. Although the machining time increased in spiral trepanning strategy, the productivity this strategy achieved promising results in terms of hole depth.

The largest hole depth achieved by spiral trepanning strategy was 22 mm depth at a scanning speed of 10 mm/s and laser power of 900 W in single pass only. In contrast, the single ring shape pattern strategy at the same combination achieved 7 mm depth with the assistance of 10 multiple passes. The interesting point about this achievement, referring Goeke and Emmelmann [19], was that a fibre laser (wavelength = 1070 nm; laser power = >1 kW; CW mode) at scanning speed of 83 mm/s could cut CFRP up to 5 mm. This result indicates the current research was able to break the threshold limit by penetrating CFRP composites of more than 5 mm depth. Hence, laser power is the most crucial factor when using spiral trepanning strategy for hole depth in laser drilling of thick CFRP.

The influence of scanning speed with the application of spiral trepanning strategy was evident through the depth and HAZ. It was observed that the HAZ decreased as the speed increased when no multi-pass cutting strategy was applied. This result was due to the shorter interaction time between the laser beam and the material. It strongly suggests the laser beam was focused with sufficient energy to vaporise the top surface of the material, and the faster movement made it less effective at cutting deeper into the material. Apart from the concern on the hole depth, a clear benefit of a spiral trepanning strategy in laser drilling of 25.4 mm-thick CFRP is the consequential reduction of thermal damage. It can be seen that the HAZ was notably reduced compared to the results in the experimental observation of the laser power, as described previously.

Furthermore, all samples had a reduction in HAZ by an average of 18% due to less interaction time. Additionally, the results show no significant appearance of matrix recession close to the hole periphery in Figure 9, which was similar to the laser power with the spiral trepanning strategy. Overall, these results indicate that scanning speed is an influential factor on the hole depth because it exceeded 7 mm. However, in terms of quality appearance, scanning speed plays a more critical role in reducing thermal damage than laser power. Consequently, this finding agrees with most results from previous research attempts.

The spiral trepanning strategy proved to be more efficient, since just one pass could penetrate deeper than the previous strategy. This strategy can be explained through the benefit of the cutting movement of spiral trepanning, where the laser beam is only focused on the interior region inside the holes with a spiral-cutting path. Furthermore, this indicates that the laser energy can be exposed on the entire cut surface, and more material can be removed by moving the laser beam spirally. Compared to the results of the single ring shape in a multi-pass strategy, the laser beam in multi-pass strategy with a single ring shape drilling operation relied on the heat dissipation, which was localised to the circumference of the hole. In turn, the laser energy reached the material as the energy flows repetitively along the same path. This effect meant the laser beam was unable to penetrate deeply or cut a large amount of material in a circular path. Conversely, spiral trepanning occurred initially at the centre of a hole and moved spirally outward inside the circular perimeter.

**Figure 11:**
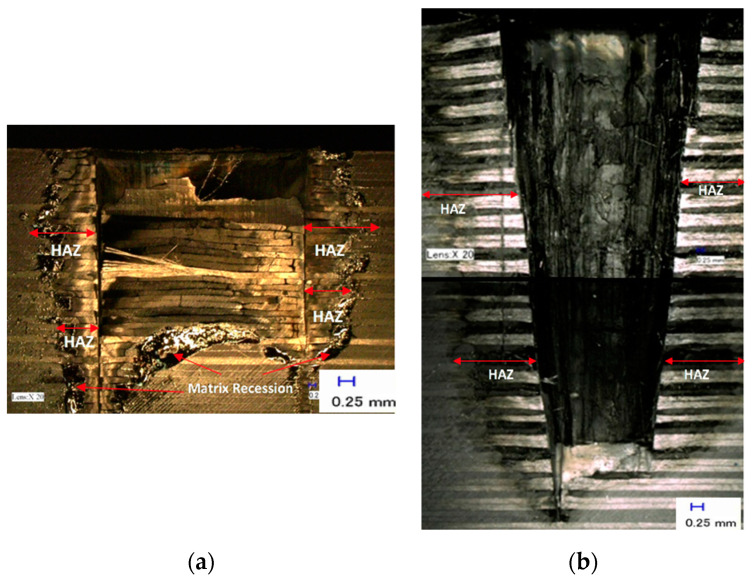
The results of two drilling strategies: (**a**) single ring shape with 10 multiple passes, and (**b**) spiral trepanning with single pass.

Figure 11 shows the comparison between a single ring shape in a multi-pass strategy and a spiral trepanning strategy. Figure 11a illustrates the single ring shape in the multi-pass strategy, with 10 passes, at laser power and scanning speed of 900 W and 20 mm/s respectively. A machining time of 27.22 s produced a hole depth of 5 mm. Meanwhile, Figure 11b shows the result of spiral trepanning in a single pass at laser power 900 W, scanning speed of 20 mm, and machining time of 18.79 s, achieved a depth of 20 mm.

The significant advantage of spiral trepanning is the achievement of a more evenly energy distribution. For a single ring shape with multi-pass cutting, high laser energy is needed for a cylindrical volume to be heated, melted, vaporised and ejected. Consequently, non-escape vaporised materials can block the laser beam from reaching the bottom of a hole, as not all fibres or plume of dust are completely ejected. Instead, the laser pumps energy into the plume, which can project heat into the surrounding material, thus increasing HAZ. Additionally, another possible cause for the deterioration of laser energy during the vaporisation is due to the materials. The by-products spread out from the holes and block the nozzle, which can affect the lens inside the cutting head. Therefore, spiral trepanning is a suitable alternative for drilling CFRP, especially for thick materials of more than 5 mm. By moving spirally, the laser beam can penetrate the materials at different locations and create a much wider cavity. This cavity reduces the risk of non-ejected materials like plume blocking the laser. At the same time, the energy can be utilised evenly in all areas of the material, resulting in higher material removal. Another advantage of this strategy is the reduction of HAZ due to the reduction of laser energy and the possible elimination of other defects. The most obvious defect in this research is matrix recession. The single ring shape in a multi-pass cutting strategy leads to significant deterioration resulting from HAZ and matrix recession, which occur because of heat localisation along the same path. Consequently, high-energy consumption was needed to vaporise the remaining fibres and make the heat dissipate towards other areas.

Carbon fibres have the highest vaporising temperature and thermal conductivity in composites that lead to increased heat dissipation into the workpiece producing a large heat zone or HAZ. The matrix is the first component of the heat, when a laser beam hits the surface. The energy needed to vaporise the fibres is typically higher than the fibre required for the matrix; a high-pressure local peak causes mechanical (delamination) damage within the compound for the short time required to vaporise a large matrix. The disadvantages of laser cutting are changes in material and decreased strength caused by common defects such as HAZ formation, kerf taper formation and reduction in cutting efficiency as workpiece thickness increases. HAZ occur when the workpiece is thermally performed, causing heat damage and material properties degradation to a certain depth or area. This event is the primary mode for transfer of energy from the surrounding material’s cutting region. The limits of HAZ are correlated with the temperature of the matrix char. In addition, the size and type of the HAZ is closely linked to the fibre orientation. The fibre orientation is related to the laser beam’s direction of travel and the fibre thermal conductivity. The perpendicular cutting of the fibres led clearly to a greater HAZ and parallel cutting had less impact on the HAZ. Unidirectional CFRP cutting by the excellent heat conductivity of carbon fibres as a result of heat channelling away from the cutting zone. The increase in cutting speed (also called feed or laser machining transverse speeds) appears to minimise the width of the HAZ. Moreover, a major difference in the size of the HAZ was observed due to the supportive gas flow, in as much that the HAZ decreases with increasing gas flow. The scale of the HAZ does not however be influenced by the form of assist gas used, like compressed air. It is evident that the main task of the help gas in laser machining of FRP is to eliminate the waste or by-products from the cutting zone by mechanical means. In addition, with increased laser power, the heat damage would be increased. The size of HAZ depends generally on thermal conductivity, cutting speed and strength of the laser beam. Thus, the spiral trepanning strategy will reduce the risk of damaging CFRP’s mechanical properties compared with single ring shape with multi-pass cutting.

Based on previous research, the major obstacle in laser drilling is to optimise the laser power during the machining process to achieve the most efficient solution in material removal and the reduction of HAZ. Consequently, when the energy is not fully utilised towards the bottom of a hole; there is a high possibility of significant taper or inclination of cylindrical volume. Further investigation identified the next potential parameter to find the optimum solution for drilling 25.4 mm-thick CFRP. In this attempt, each focal plane position (FPP) setting was chosen to understand the optics system and the explanation about the process parameters. As well as the variation of spot sizes based on the view of the window’s scale for Precitec HP1.5” cutting head, which is described in the Section 3.

**Figure 12:**
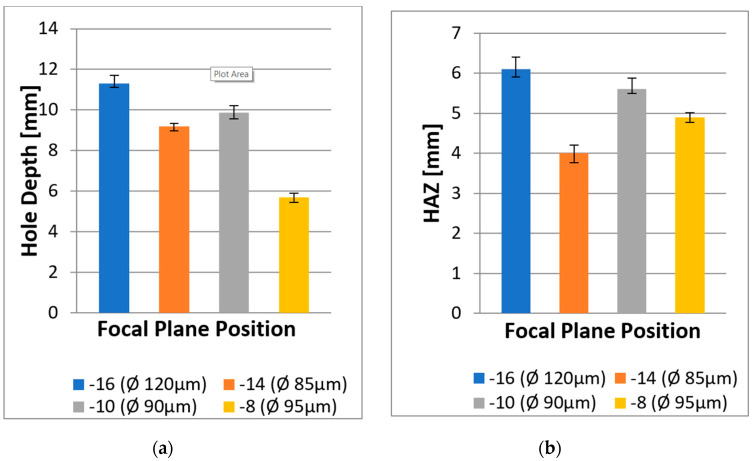
The results of two drilling strategies: (**a**) single ring shape with 10 multiple passes and (**b**) spiral trepanning with single pass.

**Figure 13:**
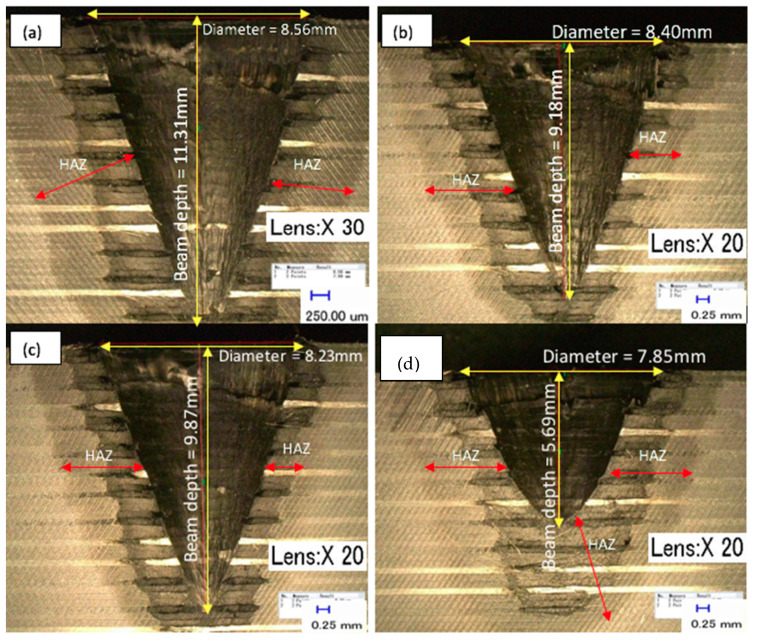
Influence of focal plane position (FPP) on hole depth and HAZ at four different view scales: (**a**) FPP: −16; (**b**) FPP: −14; (**c**) FPP: −10; and (**d**) FPP: −8.

Figure 12 shows the effects of FPP on the hole depth and HAZ. The calculation of hole depth and HAZ were repeated three times using a digital optical microscope, and all results were based on the average values. Additionally, the measurement of HAZ was covered on the radial extent of HAZ beyond the borehole walls created. Figure 13 presents the results of overall hole depth tests for different FPPs. The first image (Figure 13a) shows the FPP at −16, which created an oversized diameter of 8.56 mm. The next image (Figure 13b) illustrates the FPP at –14 with the diameter of 8.4 mm. The third image (Figure 13c) shows the result with the FPP at −10 with a diameter of 8.23 mm. The final image (Figure 13d) shows the FPP at −8 with an undersized diameter of 7.85 mm.

As can be seen, each FPP had a different spot size, which influenced the depth and thermal damage. Furthermore, the different FPP settings influenced the laser power density on the workpiece surface, specifically the changes in the induced beam spot size at the surface, which has a different effect of heat dissipation towards the material. It can also be seen that an FPP at −10 enabled the laser to produce the deepest hole, but the bottom of each hole showed significant taper or was cone-shaped. In contrast, the results at FPP of −12 with the spiral trepanning strategy resulted in a nearly-even surface and provided an excellent hole depth (i.e., 22 mm depth) and low HAZ (i.e., less than 10 mm). This result indicates that this FPP level is the most promising.

It may be that as the depth increases, the kerf width is decreased as the beam energy absorbed by the material decreases, and particularly the intensity (power per unit area) decreases as drilling progresses. The incident radiation from the keyhole, produced by decomposition from the top surface of the substance, and the growth of a vapour layer are important aspects of this experiment. The keyhole behaves as a laser beam blackbody, which limits some power as the vapour is reflected and absorbed. Additionally, the deviation of the beam from its focal point connects with the top surface, which decreases power density along the keyhole. This result is in line with Mathew et al. [13].

The level of HAZ also varied with the changing of FPP. It can be seen that FPP at −16 was the most severe, which could be caused by the spot size and the varying power intensity due to its respective size. Consequently, the larger the spot area, the more the laser power spreads over a larger area, resulting in a wider HAZ. Moreover, all FPPs produced oversized hole diameters except for FPP at −8; which could be explained by the large amount of energy at the material surface. The material area with the darkest colour of HAZ exhibited the most severe thermal damage, due to the complexities of heat propagation in CFRP, which occurred in all workpiece materials. Thus, through observing the effect of FPP with the application of a spiral trepanning strategy, the current study proves that the laser beam did not only produce a surface cut but was able to penetrate to more than 5 mm.

**Figure 14:**
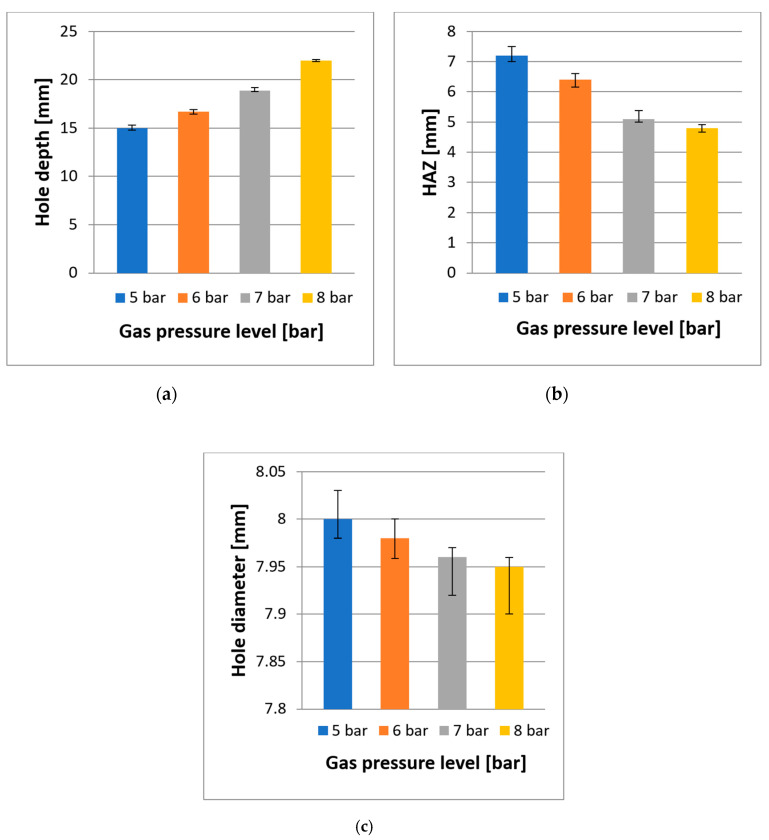
The influence of assist gas pressure on (**a**) hole depth, (**b**) HAZ, and (**c**) hole diameter.

**Figure 15:**
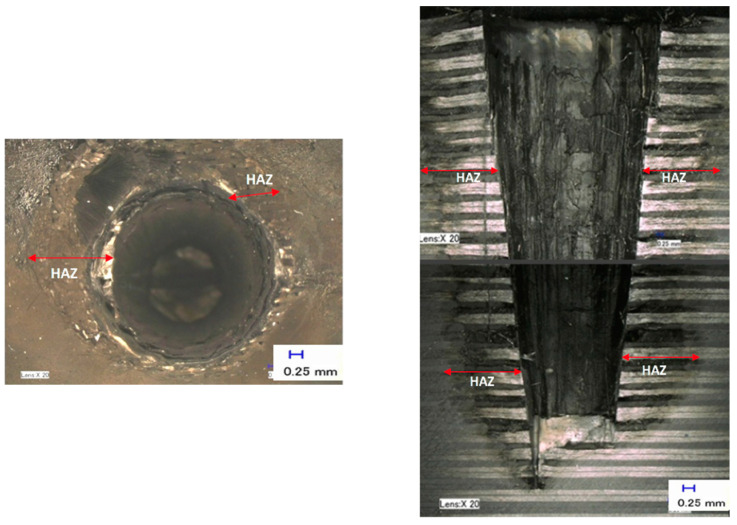
A typical example of assist gas pressure (8 bar) on overall hole depth, HAZ, and hole diameter.

The typical function of assist gas pressure in laser machining is to cool and blow away the evaporated materials and to protect the laser optics from by-products. The process parameters for this experimental study on the effect of assist gas pressure are described in the Section 3. Figure 14 shows the effect of assist gas pressure on the overall hole depth, diameter, and HAZ. Similarly, the measurements of these results were repeated three times, and the average was calculated. Figure 15 illustrates the typical outcome of assist gas pressure at a pressure level of 8 bar. The upper diagram shows the hole entry result, and the bottom one shows the effect of assist gas pressure inside the hole in a cross-section view.

As can be observed, there is a proportional relationship between assist gas pressure and hole depth, whereby increasing the assist gas pressure enables the laser beam to produce a deeper hole. The deepest hole achieved in this experiment was 22 mm at an assist gas pressure of 8 bar. This result indicates that higher pressure assists the laser beam for deep penetration, where by-products were blown away effectively, and the risk of incident radiation such as blackbody for the laser radiation was reduced. This created more room for the laser beam to penetrate deeper, allowing more material removal. Observing the bottom hole, where the laser beam achieved a flat surface, proves this.

On the contrary, the two outcomes, which were HAZ and the diameter of the holes, had inverse relationships with assist gas pressure, whereby when the gas pressure is increased, the level of HAZ decreased. However, the hole diameter changed from oversized to undersized. With low gas pressure, the material accumulated inside the hole is heated up further by the laser beam. This hot plume shields the bottom of the hole, thereby preventing the laser from cutting deeper; however, it also loses some of its heat into the surrounding borehole wall or workpiece material, which increases the temperatures the size of HAZ. This might be the reason why the top-hole diameter expanded. These explanations support the reason for the escalation of HAZ, as well as the hole depth and diameter development. As can be seen, HAZ gradually increased when the gas pressure was reduced, but no severe matrix recession occurred at the hole entry (the top view), or the cross-section view.

**Figure 16:**
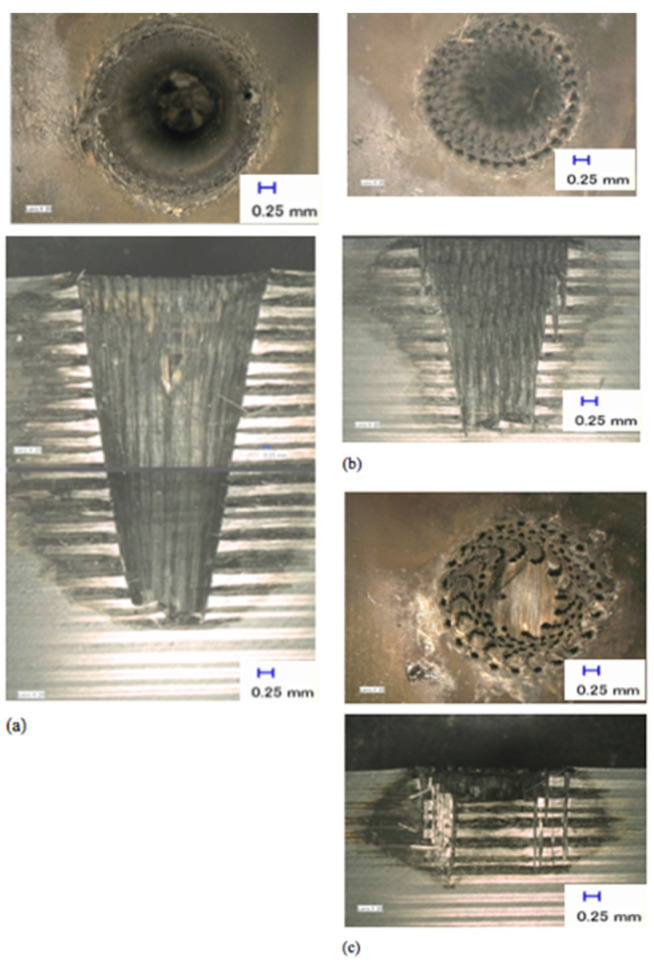
Influence of modulated laser beam on hole depth and HAZ at pulse ratio: (**a**) 10:10 ms, (**b**) 10:50 ms and (**c**) 10:90 ms.

The next experiment was conducted to study the effects of the laser beam on the depth of the holes and HAZ in modulated pulsed mode. The process parameters and explanation about the different energy delivery patterns are described in the Section 3. All holes were drilled with a spiral trepanning strategy following promising results in CW mode. Figure 16 shows the effect of the pulse ratio on the hole depth and HAZ. Three pulse ratios, which describe the ratio between ‘pulse duration’ and ‘pulse-off time’, were investigated: 10:10, 10:50, and 10:90 ms. These three ratios had a pulse repetition rate (PRR) of 50, 16.7, and 10 Hz, respectively. Figure 16a shows the outcome at the pulse ratio of 10:10 ms, which produced the deepest hole in modulated pulsed mode at 17 mm. The cross-section was divided into two images because the microscope could only record the highest view range, which is the maximum overall view. Figure 16b,c shows the outcomes at the pulse ratios of 10:50 and 10:90 ms, respectively. All measurements were repeated thrice, and the average values were obtained.

**Figure 17:**
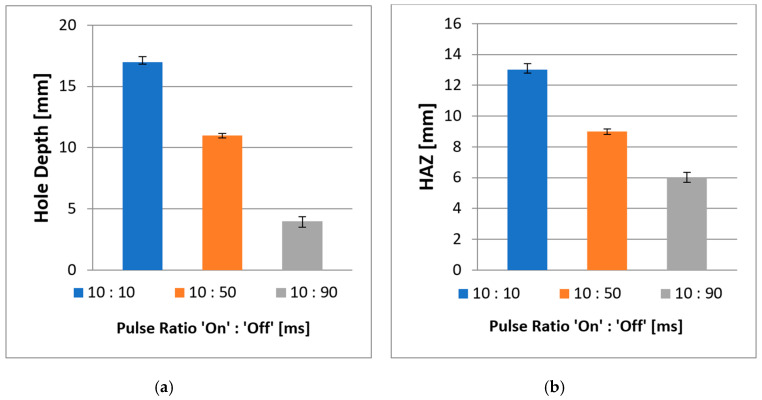
Effect of a modulated laser beam on (**a**) hole depth and (**b**) HAZ.

Figure 17 shows the inverse relationships between the pulse ratio and HAZ, as well as the depths of the holes, which show that as the pulse-off time increases, both HAZ and hole depths decreased. None of these cases led to the full penetration of the material as expected. However, the results in Figure 16 and Figure 17a show that with a pulse ratio of 10:10 ms, the laser could penetrate the material, even with less laser energy per unit. Furthermore, a PRR of 50 Hz provided significant material removal and was able to penetrate the material with a depth of 17 mm. Figure 16b shows that a PRR of 16.7 Hz produced a significant amount of grooving materials left inside the holes or the indentations on the edge of the surface. The laser could not wholly remove the uncut material because the off duration was too long and the laser only shot individual holes into the material rather than creating a (semi)-continuous cut line. Consequently, by shooting the laser in pulse/discontinuous mode, the amount of energy pumped into the workpiece material was significantly reduced.

There were no significant oversized or undersized holes at the top surface in this experimental study; however, a PRR of 10 Hz in Figure 16c was unsuccessful in removing a significant amount of materials, which appeared on the top surface with some roundish shapes, although some appeared to be somewhat distorted. The distortion was probably due to the reduced amount of energy that went into the materials, which created many craters. Apart from the overall hole depth, by using a modulated laser beam, the HAZ was smaller, and the matrix recession at the hole entry side and the internal holes showed no severe damage compared to CW laser mode due the cooling time between the pulses. Thus, based on both figures, a high PRR at 50 Hz (pulse ratio 10:10) is more advantageous for hole depth (i.e., achieved 17 mm depth). Additionally, a low PRR of 16.7 and 10 Hz at pulse ratios of 10:50 and 10:90, respectively, lead to a considerable reduction of HAZ (i.e., 31% and 54% for pulse ratios of 10:50 and 10:90, respectively) compared to a PRR of 50 Hz, which obtained the average length of 13 mm.

## 5. Conclusions and Future Work

The work discussed in this article concerned the use of a 1 kW fibre laser machine for laser drilling of 25.4 mm-thick carbon fibre reinforced polymer composites (CFRP). Several conclusions can be drawn based on the following observations:

(a) The overall experiments in laser drilling of thick CFRP were unsuccessful in fully penetrating the 25.4 mm-thick CFRP, even though the experiments were conducted with various machining parameters. However, the experimental results demonstrated the feasibility of using the IPG single-mode 1 kW fibre laser to penetrate CFRP composites at a depth of up to 22 mm;
(b) For a single ring shape with multi-pass cutting, high laser energy is needed for a cylindrical volume to be heated, melted, vaporised and ejected. Consequently, non-escape vaporised materials can block the laser beam from reaching the bottom of a hole because not all fibres are ejected entirely, and there is a strong possibility that the remaining fibres are inside the hole. Conversely, for a spiral trepanning strategy, the laser beam only focuses on the interior region of the entire hole, with a spiral-cutting path; this indicates that the laser energy can be optimised, and more material may be removed by moving the laser beam spirally;
(c) Experimentally, it was found that laser power, assisted gas pressure, FPP, pulse ratio were the most influenced parameters for affecting depth of cut, by adapting the spiral trepanning drilling strategy. Scanning speed and pulse ratio were the main parameters affecting HAZ, matrix recession and other defects—also with the application of a spiral trepanning strategy;
(d) The spiral trepanning strategy penetrated 80% of the total thickness of the CFRP in CW mode, whereas in a modulated laser beam, i.e., laser pulse mode only penetrated 67%;
(e) The modulated laser pulse mode could be used in deep-hole drilling. Furthermore, there was a positive outcome in the modulated beam mode results compared to other experimental results, showing that HAZ can be significantly reduced. With high beam intensity and better focusing of modulated fibre laser beam, it shows a smaller heat distribution, i.e., thermal load, during the drilling process. This assists in drilling the CFRP composites with minimal damages. However, the limitation was only the reduction of laser energy distribution, which leads to significant reduction of hole depth due to modulated pulsed mode, i.e., pulse ratio. Pulse ratio is the crucial factor for the depth of cut, which can affect the laser power due to higher pulse off-time. For the current research, the modulated laser pulse mode could achieve full penetration by implementing a multi-pass cut with a spiral trepanning strategy or by drilling from both sides without the multi-pass.

Further research to determine the temperature distribution within the CFRP composites should be undertaken. The interaction between the laser and the fibre matrix’s microstructure in the material will be important to explore; Xu et al. [20] created an optical absorption model based on this interaction by incorporating the effect of wavelength control, fibre volume fraction, and power intensity distribution within the laser spot. They performed experiments on CFRP samples via the Nd: YVO_4_ laser milling process and found that the best approach for the machining process used a short wavelength and arbitrarily polarised laser source. This could be useful to research the laser spot, as it is normally as big as many fibres. The arbitrary polarised laser will probably remove the influence of variable fibre orientation in CFRP for thick samples in particular. The absorption model can provide a good solution by applying the spiral trepanning technique for assessing the absorptiveness and substantial reductions in HAZ, delamination or other damage in future experiments.

## Figures and Tables

**Table 1:** Critical laser machining parameters.

Parameter
Type of Laser
Laser Mode Setting
Laser Power
Scanning Speed
Type of Assist Gas
Gas Pressure
Nozzle Diameter
Stand-Off Distance
Focal Plane Position (FPP)
Focal Length
Focal Lens Diameter
Beam Spot Diameter

**Table 2:** Properties of the material sample.

Material	Grade	Yield Strength	Density
CFRP (carbon fibre reinforced polymer)	M21	835 MPa	2.06 g/cm^3^

**Table 3:** Parameters for laser spiral trepanning strategy.

Parameter	Parameter Input Value/Setting
Laser Mode Setting	Continuous-wave (CW)
Laser Power	Constant: 900 W
	Variation: 900, 700, 600, 400 W
Scanning Speed	Constant: 10 mm/s
	Variation: 20, 30, 40, 50 mm/s
Type of Assist Gas	Argon
Gas Pressure	8 bar
Nozzle Diameter	1 mm
Stand-Off Distance	1 mm
Focal Plane Position (FPP)	−12 mm
Focal Length	7.5′
Focal Lens Diameter	1.5″
Beam Spot Diameter	70 µm (at reference point, FPP = −12 mm)

**Table 4:** Parameters for laser drilling with pulse mode operation.

Parameter	Parameter Input Value/Setting
Laser Mode Setting	Modulated pulse
Laser Power	900 W
Scanning Speed	10 mm/s
Pulse Duration	10 ms
Pulse-Off Time	10, 50 and 90 ms
Pulse Repetition Rate (PRR)	50, 16.7 and 10 Hz
